# Phloem Girdling of Norway Spruce Alters Quantity and Quality of Wood Formation in Roots Particularly Under Drought

**DOI:** 10.3389/fpls.2018.00392

**Published:** 2018-03-27

**Authors:** Gina Rainer-Lethaus, Walter Oberhuber

**Affiliations:** Department of Botany, University of Innsbruck, Innsbruck, Austria

**Keywords:** carbon allocation, drought, girdling, Norway spruce, root growth, wood anatomy

## Abstract

Carbon (C) availability plays an essential role in tree growth and wood formation. We evaluated the hypothesis that a decrease in C availability (i) triggers mobilization of C reserves in the coarse roots of *Picea abies* to maintain growth and (ii) causes modification of wood structure notably under drought. The 6-year-old saplings were subjected to two levels of soil moisture (watered versus drought conditions) and root C status was manipulated by physically blocking phloem transport in the stem at three girdling dates (GDs). Stem girdling was done before the onset of bud break [day of the year (doy) 77], during vigorous aboveground shoot and radial stem growth (GD doy 138), and after cessation of shoot growth (GD doy 190). The effect of blockage of C transport on root growth, root phenology, and wood anatomical traits [cell lumen diameter (CLD) and cell wall thickness (CWT)] in earlywood (EW) and latewood (LW) was determined. To evaluate changes in belowground C status caused by girdling, non-structural carbohydrates (soluble sugars and starch) in coarse roots were determined at the time of girdling and after the growing season. Although fine root mass significantly decreased in response to blockage of phloem C transport, the phenology of root elongation growth was not affected. Surprisingly, radial root growth and CLD of EW tracheids in coarse roots were strikingly increased in drought-stressed trees, when girdling occurred before bud break or during aboveground stem growth. In watered trees, the growth response to girdling was less distinct, but the CWT of EW significantly increased. Starch reserves in the roots of girdled trees significantly decreased in both soil moisture treatments and at all GDs. We conclude that (i) radial growth and wood development in coarse roots of *P. abies* saplings are not only dependent on current photosynthates, and (ii) blockage of phloem transport induces physiological changes that outweigh drought effects imposed on root cambial activity and cell differentiation.

## Introduction

Norway spruce [*Picea abies* (L.) Karst.] is the dominant coniferous tree species in the Central European Alps. Natural populations of *P. abies* are found from the lower montane region up to the tree line (Schmid-Vogt, 1977; Ellenberg and Leuschner, 2010), indicating its ability to acclimate to different site and climatic conditions. However, due to the predominant occurrence of fine roots in the upper soil horizon (Puhe, 2003), *P. abies* shows high sensitivity to drought stress, leading to reduced growth, a shortened growing season, and early culmination of radial stem growth in late spring (Mäkinen et al., 2001; Alavi, 2002; Pichler and Oberhuber, 2007; Lévesque et al., 2013; Swidrak et al., 2014). Fine roots are defined as short-lived, small-diameter (≤ 2 mm) non-woody roots that are responsible for water and nutrient absorption in plants (Wells and Eissenstat, 2003; Rytter, 2013). It is well known that due to high turnover, fine roots depend heavily on the import of new carbon (C) from the canopy (Jackson et al., 1997; Matamala et al., 2003; Gaul et al., 2009). According to the balanced-growth hypothesis, which suggests that the plant will preferentially allocate biomass to the organ that is capturing the resource limiting growth (Shipley and Meziane, 2002), plants experiencing water shortage allocate more biomass belowground (Mokany et al., 2006; Poorter et al., 2012; Hagedorn et al., 2016). Although a significant increase in the root-shoot ratio in response to periodic drought treatment was reported for *P. abies* seedlings (Sonesson and Eriksson, 2003), the degree of change in the root-shoot ratio is affected by the intensity of soil drought (Schall et al., 2012), because suppressed root growth and increase in fine root mortality in response to drought stress is a frequently reported phenomenon (e.g., Gaul et al., 2008; Brunner et al., 2009; Rytter, 2013; for a review see Brunner et al., 2015).

In the course of secondary growth, trees are able to adjust their water transport systems to changing water availability (for a review see Fonti et al., 2010; Bittencourt et al., 2016). For example, under drought stress xylem conduit dimensions frequently decrease while conduit walls are reinforced (e.g., Dünisch and Bauch, 1994; Arnold and Mauseth, 1999; Martin-Benito et al., 2013), i.e., smaller cells with thicker cell walls are built to allow maintenance of an intact water transport system to avoid downregulation of photosynthesis and/or xylem cavitation (Hacke et al., 2001; Bréda et al., 2006; Rennenberg et al., 2006). Wood formation is also influenced by C availability, because C compounds provide energy for cambial activity (cell division), produce water turgor pressure during expansive growth (cell enlargement) and provide polysaccharides during structural growth (cell wall thickening) (Gessler et al., 2009; Muller et al., 2011; Simard et al., 2013; Deslauriers et al., 2016). Under prolonged drought stress C allocation to cell wall formation is diminished leading to formation of ‘light rings’ (Liang and Eckstein, 2006; Martin-Benito et al., 2013; Balducci et al., 2015). Accordingly, C and water availability influence xylem anatomy and hydraulic function through effects on cell enlargement and cell wall thickening. However, studies on the responses of the water transport system in roots to changes in C and water availability are scarce (Brunner et al., 2015).

Trees have substantial amounts of non-structural carbohydrate (NSC) reserves (mainly starch and soluble sugars; Hoch et al., 2003), which may be available to act as mobilizable safety reserves for respiration, growth, and wood formation in case of reduced photosynthetic C supply (Regier et al., 2009; Hartmann et al., 2013). Physical blockage of C transport through girdling immediately terminates the supply of current photosynthates to the roots while enabling water transport in the reverse direction through the xylem. This manipulative type of treatment is useful to study the impact of C availability on tree growth (e.g., Maier et al., 2010; Maunoury-Danger et al., 2010; De Schepper and Steppe, 2011; López et al., 2015; Oberhuber et al., 2017). Manipulation of the root C status by disruption of phloem transport in the stem under high vs. low soil moisture content can reveal (i) the ability of roots to mobilize C reserves for root growth, and (ii) how it impacts the tracheid anatomy of the new conduits formed. Girdling applied at different phenological stages, i.e., before, during, and after stem growth acts as a C sink, can reveal the dependency of root growth and wood development on current photosynthates throughout the growing season.

The focus of this study therefore was to determine the effects of interrupted stem C flow at distinct phenological stages on root growth and xylem anatomical traits in *P. abies* saplings under different levels of soil water availability, i.e., watered vs. drought-stressed. We hypothesized that blockage of C transport impairs root growth (elongation and radial growth) and modifies tracheid differentiation, resulting in a decrease in CLD and CWT, even under non-water-stressed conditions. Furthermore, because phloem girdling also inhibits transport of shoot-derived growth substances (especially auxin) necessary for growth and development of roots (Ljung et al., 2001; Fu and Harberd, 2003; Takahashi, 2013), we expected no root growth when girdling occurred before bud break and the onset of aboveground growth. This study contributes to a developing understanding of the early timing of maximum radial stem growth found in several coniferous species exposed to drought at the start of the growing season (Oberhuber et al., 2014), and the interaction between C availability and drought on wood formation.

## Materials and Methods

### Plant Material and Treatments

The study was conducted at the Botanical Garden of the Department of Botany (University of Innsbruck, Austria) in 2015 using 6-year-old *P. abies* trees with a mean stem height and diameter of 1.3 m and 3.5 cm, respectively. Saplings were grown in 80-l containers that were filled with a well-draining potting mixture fertilized with calcium ammonium nitrate containing 27% N (NAC 27 N; Borealis L.A.T., AT) and a drainage layer at the bottom of the container. A uniform microclimate was ensured by placing the containers in a regular polytunnel covered with clear polythene (200 micron UV stabilized film). Before starting the experiment, trees were allowed to recover from transplant shock for one complete growing season (October 2013 through March 2015).

Two levels of soil moisture were applied by irrigating trees weekly to field capacity in the early morning (watered treatment) or by watering in 10- to 14-day-intervals with 50% of the amount of irrigation used for watered trees (drought treatment). Trees were divided into the control subset (no phloem blockage) and three subsets of phloem blockage treatment at different girdling dates [GDs; day of the year (doy) 77, doy 138, and doy 190]. In total, 80 trees were included in the study: 2 soil moisture treatments × 4 subsets × 10 trees per subset = 80 trees.

Air temperature, relative air humidity, and solar radiation (PAR) were recorded at two meters’ height within the polytunnel throughout the study period in 2015 (CS215 temperature and relative humidity sensor, LI-200S Pyranometer Sensor; Campbell Scientific, Shepshed, United Kingdom). Volumetric soil water content (SWC) and soil temperature were measured in the uppermost 30 cm and at 10 cm soil depth, respectively, in both soil moisture treatments (*n* = 10 per treatment; T 107 Temperature Probe, and CS616 Water Content Reflectometer; Campbell Scientific, Shepshed, United Kingdom). Environmental data were collected every minute and averaged in 30-min intervals using a CR1000 data logger and AM 16/32 multiplexers (Campbell Scientific, Shepshed, United Kingdom).

### Manipulation of C Availability by Phloem Blockage

To accomplish blockage of C transport from leaves to roots, we applied double girdling by detaching two 1- to 2-cm-wide bands of bark (extending to the xylem) at stem heights of *c.* 5 and 15 cm (*cf.* De Schepper et al., 2010; Oberhuber et al., 2017). Dehydration of xylem tissue was prevented by covering girdling zones with aluminum foil. Based on phenological stages determined in a previous study (Swidrak et al., 2013), trees were girdled (i) several weeks before bud swelling and onset of cambial activity in mid-March (GD doy 77), (ii) during shoot growth and earlywood (EW) formation in mid-May (GD doy 138), and (iii) after aboveground growth of the terminal shoot ceased in July (GD doy 190).

### Determination of Root Growth and Wood Anatomical Parameters

At the end of the study period in late October, cross-sections with a diameter of *c*. 1 cm were collected from coarse roots that had developed ≥ 5 cm below the soil surface. Radial growth was measured on transverse sections of *c*. 20 μm thickness that were cut using a rotary microtome and stained with a water solution of 0.05% cresyl fast violet. Ring widths were measured to the nearest 0.01 mm by applying the image analysis software ProgRes Capture Pro (version 2.8.8, Jenoptik, Jena, Germany). Due to the occasional occurrence of eccentric diameter growth, mean radial root growth was calculated from 5 radii per cross-section and 10 trees per treatment (i.e., soil moisture and GD). Dating of the growth zones was accomplished visually by cross-dating time series, i.e., matching year-to-year variations in ring width among different trees (Cook and Kairiukstis, 1990). To enable comparison of the effects of girdling between the control and girdled trees, the radial growth of each tree was standardized using the ring widths of the year prior to girdling (Rossi et al., 2003). In every sample, ring widths of the previous three years were measured and used to correct inherent differences in radial root growth among trees. Ring width was corrected as follows:

$${\text{r}}_{\text{cwi}}={\text{rw}}_{\text{msi}}\times {\text{rw}}_{\text{mi}}/{\text{rw}}_{\text{si}}$$

where r_cwi_ = corrected ring width, rw_msi_ = measured ring width, rw_mi_ = mean ring width of previous rings of all i-samples, and rw_si_ = ring width of previous rings for each i-sample.

The same transverse sections used for measuring radial root growth were used to determine wood anatomical parameters. CLD and CWT were measured along five separate cell rows throughout five EW and LW cells, i.e., *n* = 25 cells per tree (*n* = 5 trees). Mean values and standard deviations (SDs) were calculated from *n* = 125 measurements per subset (GD and soil moisture treatment). The ratio between CLD and CWT defined the proportion of cell-wall material. Statistically significant differences among control and girdled trees with respect to these cell parameters were determined by applying Student’s independent sample *t*-test.

Root elongation growth was determined by applying the non-destructive minirhizotron technique (Vamerali et al., 2012), which allows a continuous study of root growth during the growing season. Clear acrylic tubes with an inner diameter and length of 6.3 and 50 cm, respectively, were inserted at 45–50° angles in 10 containers for each environmental setting of controls (2 treatments × 10 replications = 20 tubes) and in drought-stressed girdled trees (three GDs × 10 replications = 30 tubes). The aboveground end of the tube was wrapped with white plastic tape to minimize solar heating and to prevent light from entering the tube. The tube–soil interface was allowed to equilibrate for one growing season before imaging to avoid artifacts (Johnson et al., 2001). Images were collected from March (doy 77) through October (doy 278) in 4-week intervals (CI-600, CID Bio-Science, Inc., Camas, WA, United States), when no soil frost was recorded.

Following collection, images were analyzed for the onset and end of root elongation using image analysis software (ImageJ, 1.37, National Institutes of Health, Bethesda, MD, United States). Newly formed roots are unsuberized and have a whitish appearance (Pallardy, 2008). Therefore, onset of root elongation was defined when white root tips were seen for the first time between two succeeding observation dates. Accordingly, cessation of root elongation was determined when no change in root elongation was seen between succeeding observation dates. Quantitative analysis of root elongation growth was not possible because identical placement of the observation tube on the root ball could not be achieved in each pot. The belowground biomass (dry weight) of fine roots with a diameter ≤ 2 mm was measured at the end of the experimental period (late October) for all trees (control and girdled trees). To this end, trees were removed from containers, the rootstock was separated and fine roots were detached by hand. Fine roots remaining in the containers were collected by sieving. Due to coverage of the soil surface with plastic film, fine roots of herbaceous species were not present. Dead fine roots, which were assessed by color, elasticity, and toughness, were discarded. Total live fine root biomass of each tree was weighed ( ± 0.01 g) after drying at 65°C for 72 h.

### Determination of Non-structural Carbohydrates (NSCs)

Measurements of starch and soluble sugars (sucrose, glucose, fructose) were performed on watered and drought-stressed trees (*n* = 10 trees per treatment) using bark samples collected from coarse roots at the end of the study period. This approach was justified because (i) bark samples contain up to 10-fold higher NSC concentrations compared to xylem (Landhäusser and Lieffers, 2003; Gruber et al., 2013), (ii) a close coupling of phloem and xylem NSC reserves exists (Van Bel, 1990), and (iii) we were primarily interested in changes in NSC content and composition caused by blockage of phloem C transport rather than determination of total root NSC reserves. The samples were collected in the morning to minimize the effects of diurnal NSC changes and were denatured within 1 h by heating in a microwave at 600 W for 90 s (Hoch et al., 2003). Thereafter, the samples were dried at 60°C to a constant weight and ground into powder (Tissuelyser II, Qiagen, Germany). Polyvinylpyrrolidone (PVP, 0.5 mg) was added to *c*. 40 mg of finely ground plant material to bind phenolic substances. Ethanol (40% v/v, 15 min at 60°C) was used to extract soluble carbohydrates from the samples. Enzymatic conversion of the soluble carbohydrates and photometric determination of NSCs were performed using a semiautomatic system for photometric testing (Rida Cube Scan analyzer, R-Biopharm, Darmstadt, Germany) and the corresponding enzymatic kits (for further details see Oberhuber et al., 2017).

## Results

**FIGURE 1:**
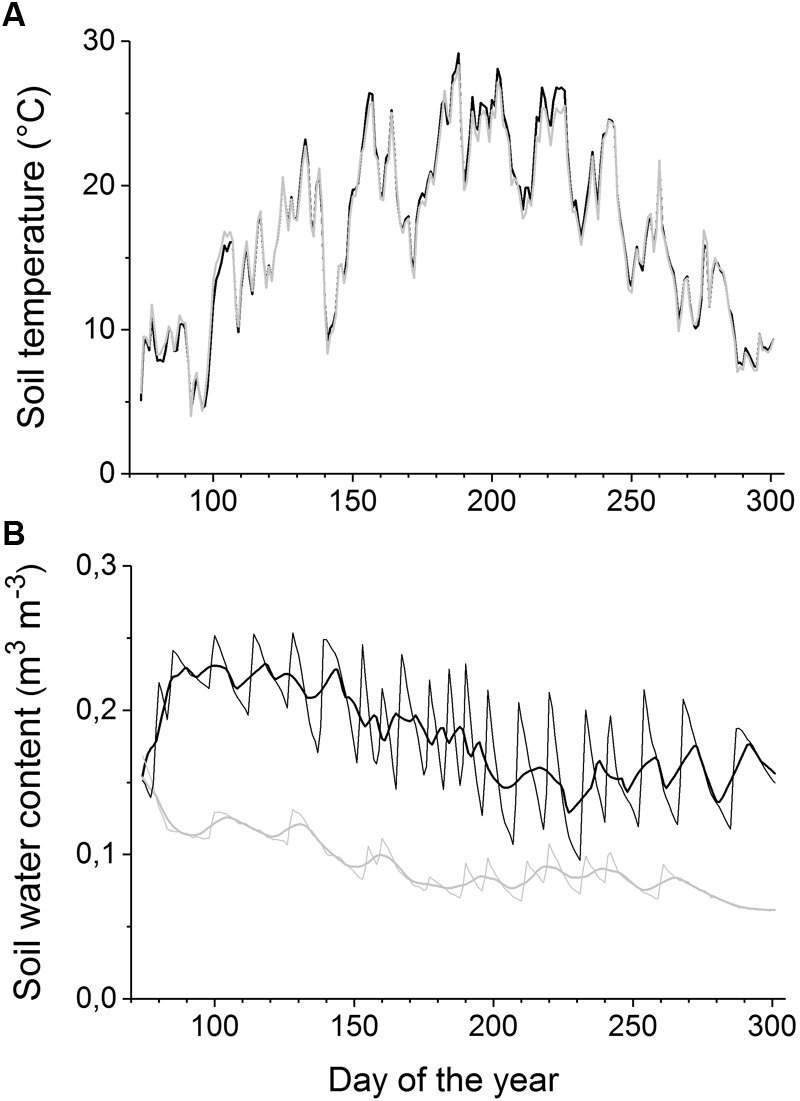
**(A–B)** Soil temperature **(A)** and soil water content **(B)** during the growing season 2015 in watered (black lines) and drought-stressed trees (gray lines). For soil water content, daily means (thin lines) and 10 days moving averages (thick lines) are shown.

The mean daily soil temperature during April through October was 18.4 ± 5.3°C (both soil moisture treatments; **Figure 1A**). Mean daily air temperature and relative humidity during the study period were 18.1 ± 5.6°C and 65.5 ± 12.8%, respectively, and the mean daily maximum solar radiation was 1005 μmol m^-2^ s^-1^ (data not shown). At the study’s commencement, the volumetric SWC averaged 0.14 ± 0.02 and 0.15 ± 0.02 m^3^ m^-3^ in the watered and drought-stressed treatments, respectively, and the mean growing season SWC values were 0.09 ± 0.02 m^3^ m^-3^ in the drought-stressed and 0.18 ± 0.04 m^3^ m^-3^ in the watered containers (**Figure 1B**).

**FIGURE 2:**
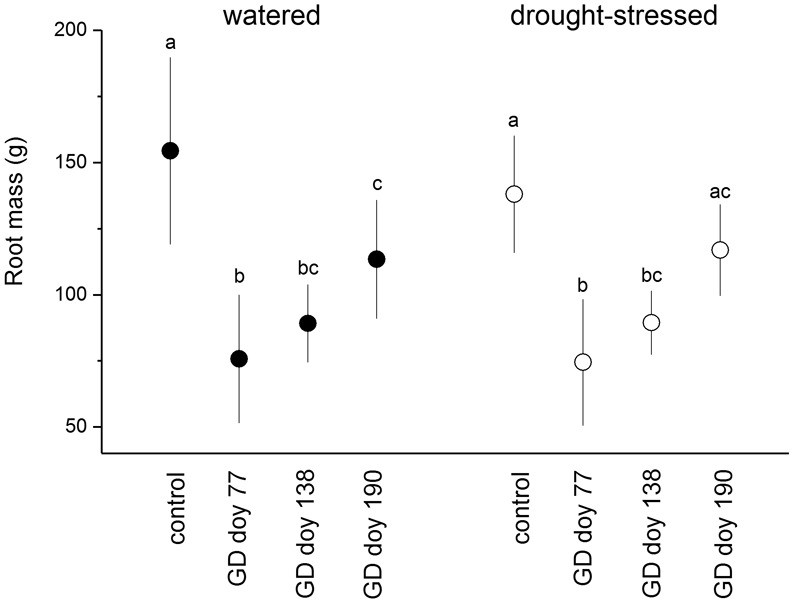
Fine root mass (diameter ≤ 2 mm) of watered and drought-stressed trees (filled and open circles, respectively) at the end of the study period. Significant effects of girdling within a soil moisture treatment are indicated by different letters (*P* ≤ 0.01; Student’s independent sample *t*-test). Bars indicate standard deviations.

In response to girdling, the fine root biomass (≤ 2 mm) in both soil moisture treatments was significantly reduced compared to controls irrespective of GD, except in drought-stressed trees at GD doy 190 (**Figure 2**). The fine root biomass of the control and girdled trees did not differ significantly among soil moisture treatments (*P* > 0.05).

The drought-stressed treatment control and girdled trees showed onset and end of root elongation growth between May 19 and June 18, and August 13 and September 4, respectively (for example root scan images see Supplementary Figure S1). Time of girdling had no effect on the phenology of root elongation growth in drought-stressed trees. In watered trees (controls), root growth started earlier (between April 21 and May 19) and ended later (between September 4 and October 5). In response to girdling before growth onset (GD doy 77), all girdled trees died between late August and mid-September, irrespective of water availability. Although all drought-stressed trees girdled in mid-May (GD doy 138) had died by the end of September, watered trees girdled on the same date were less affected (only four trees showed intensive needle browning in October). No tree mortality was observed in controls and trees girdled in July (GD doy 190).

**FIGURE 3:**
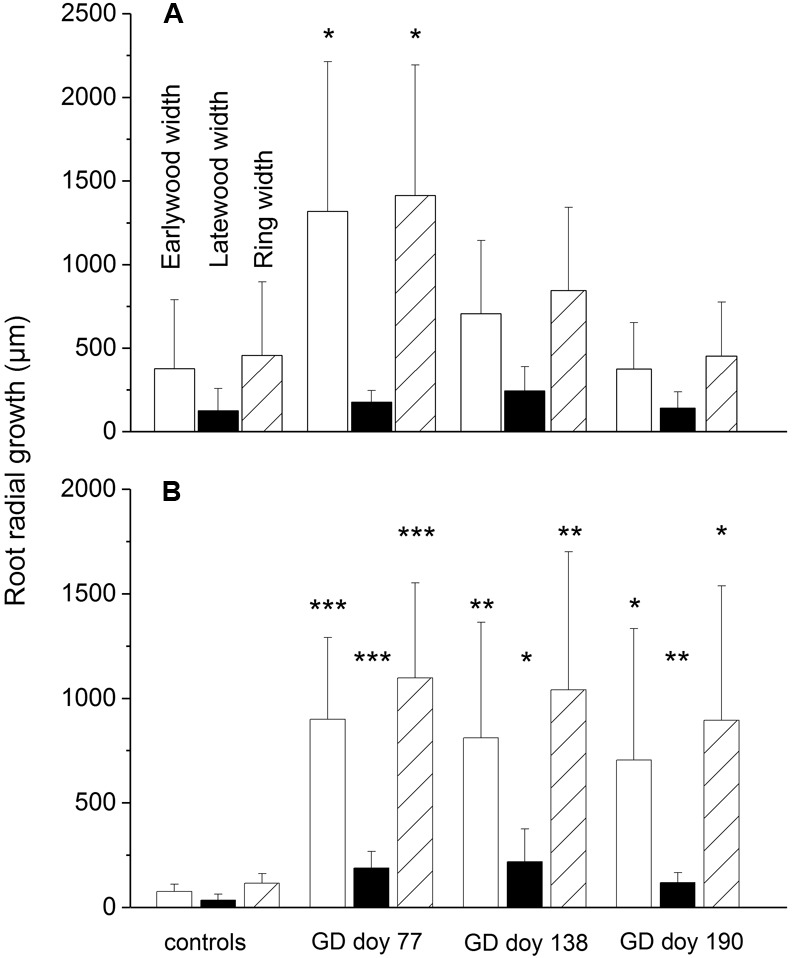
**(A–B)** Radial growth of coarse roots in response to girdling in watered **(A)** and drought-stressed trees **(B)**. Bars indicate standard deviations. Asterisks indicate statistically significant differences between girdled trees and controls (^∗^*P* ≤ 0.05; ^∗∗^*P* ≤ 0.01; ^∗∗∗^*P* ≤ 0.001; Student’s independent sample *t*-test). Note that the axes have different scales.

In the drought-stressed treatment, blockage of phloem C-transport induced a significant increase in radial root growth [EW width, latewood (LW) width and total ring width] compared to controls at all GDs, whereas in watered trees only girdling before growth onset (GD doy 77) induced a significant increase of EW width and total ring width (**Figure 3**). Ring width and EW width of the non-girdled trees differed significantly among soil moisture treatments (*P* ≤ 0.01). The annual increments and anatomical parameters of coarse roots in the year before the experiment were not significantly different among the subsets, except for LW width in the drought-stressed subset girdled at GD doy 77 in the experimental year (Supplementary Table S1).

Analyses of wood anatomical parameters in coarse roots primarily revealed that CWT in watered trees and CLD in drought-stressed trees were significantly larger in EW in response to girdling (**Table 1**). Correspondingly, the ratio of CLD: CWT in EW was significantly decreased at GD doy 138 and doy 190 in watered trees and significantly increased at GD doy 77 and doy 138 in drought-stressed trees compared to controls. The anatomical parameters of the LW did not significantly respond to girdling, except for an increase in CWT after GD doy 77 under drought (*P* ≤ 0.05) leading to a significant decrease in CLD: CWT (*P* ≤ 0.01) compared to controls.

**Table 1:** Wood anatomical parameters of earlywood (EW) and latewood (LW) in coarse roots in controls and watered and drought stressed girdled trees (CWT = cell wall thickness; CLD = cell lumen diameter).

	watered control	GD doy 77	GD doy 138	GD doy 190	drought control	GD doy 77	GD doy 138	GD doy 190
CWT (μm)					
EW	2.8 ± 0.2	3.3 ± 0.2**	3.2 ± 0.1**	3.4 ± 0.2**	3.5 ± 0.3	3.7 ± 0.1	3.2 ± 0.2	3.8 ± 0.3
LW	4.3 ± 0.4	4.8 ± 0.5	4.7 ± 0.6	4.9 ± 0.5	4.3 ± 0.6	5.7 ± 0.5*	4.6 ± 0.4	4.8 ± 0.4
CLD (μm)					
EW	18.0 ± 1.4	19.5 ± 1.3	17.9 ± 1.5	17.2 ± 0.8	14.7 ± 1.5	22.0 ± 1.5***	20.4 ± 1.5***	15.5 ± 1.5
LW	6.2 ± 0.8	5.6 ± 2.0	5.6 ± 0.9	4.2 ± 1.0	6.1 ± 1.4	4.5 ± 0.5	6.6 ± 1.2	5.2 ± 1.4
CLD:CWT					
EW	6.6 ± 0.7	5.9 ± 0.8	5.6 ± 0.5*	4.5 ± 0.4***	4.2 ± 0.8	6.0 ± 0.4**	6.4 ± 0.4***	4.1 ± 0.7
LW	1.4 ± 0.2	1.2 ± 0.3	1.2 ± 0.2	0.8 ± 0.2**	1.4 ± 0.2	0.8 ± 0.1**	1.4 ± 0.2	1.1 ± 0.2

*Asterisks indicate significant effects of girdling within a soil moisture treatment (^∗^*P* ≤ 0.05; ^∗∗^*P* ≤ 0.01; ^∗∗∗^*P* ≤ 0.001).*

**FIGURE 4:**
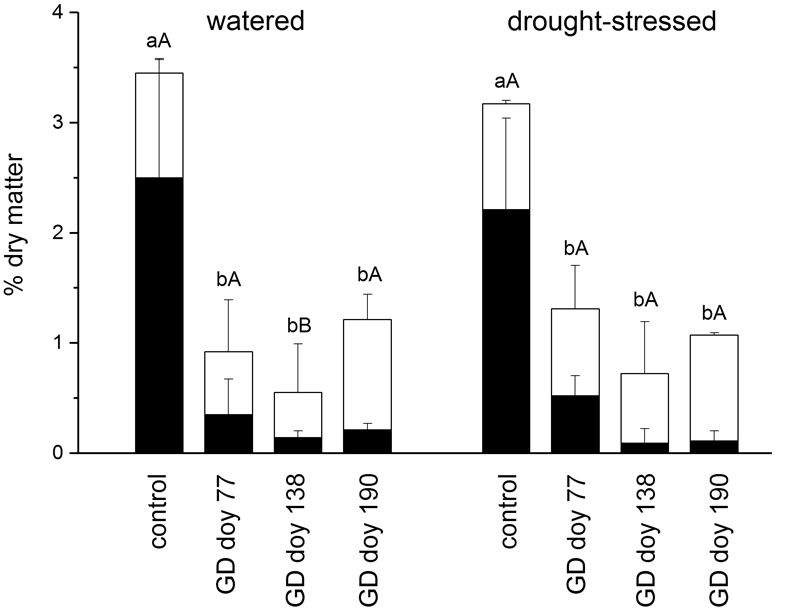
Mean concentration of starch (black bars) and soluble sugar (open bars) at the end of the study period in the coarse root of watered and drought-stressed trees. Significant effects of girdling within a soil moisture treatment are indicated by different letters (Mann–Whitney *U*-test; lower case letters: starch, *P* ≤ 0.001; upper case letters: soluble sugar, *P* ≤ 0.01). Bars indicate standard deviations.

The main outcome of girdling on the NSC content in coarse roots was a highly significant decrease in starch content irrespective of soil moisture treatment and GD, whereas soluble sugars were nearly always not significantly different from controls in both soil moisture treatments (**Figure 4**). Total NSCs were significantly different from controls at all GDs in both soil moisture treatments (*P* ≤ 0.001). The NSC content (starch and soluble sugars) of the control and girdled trees did not differ significantly among soil moisture treatments (*P* > 0.05).

## Discussion

### Effects of Drought and Modified C Availability on Radial Root Growth and Tracheid Differentiation

The main objective of this work was to study the effects of phloem blockage at different phenological stages on root growth and wood anatomy in *P. abies* saplings exposed to different soil water availability. Several authors reported accumulation and depletion of NSC above and below the girdling zone, respectively, leading to stimulation (above girdling) and cessation of radial stem growth (below girdling; Noel, 1970; Högberg et al., 2001; Daudet et al., 2005; Maunoury-Danger et al., 2010; De Schepper and Steppe, 2011; Oberhuber et al., 2017).

Contrary to our expectations, we observed a significant increase in radial growth in coarse roots in response to girdling (**Figure 3**) at the expense of starch reserves (**Figure 4**). These results indicate that girdling triggers degradation of starch to support radial root growth particularly under drought. Similarly, Jordan and Habib (1996) reported NSC depletion in roots of young peach trees due to girdling and Hartmann et al. (2013) found that reduced phloem function caused by lethal drought led to carbohydrate depletion in *P. abies* roots. The mean age of C used to grow new roots in temperate forests was found to be < 1–2 years (Gaudinski et al., 2009; Endrulat et al., 2010) indicating that phloem blockage provokes utilization of stored C reserves for radial root growth. Overall, growth data determined below the girdling zone revealed that aboveground (*cf.* Winkler and Oberhuber, 2017) and belowground cambia (this study) located below the girdling zone responded differently to physical blockage of phloem transport. Whereas shoot-derived auxin transported through the phloem with the sap flow might be necessary for radial stem growth to occur, it was not required for the initiation of root growth in girdled trees (Supplementary Figure S1).

The consistently measured low NSC content in coarse roots at the end of the growing season in dead girdled trees is consistent with findings of Jordan and Habib (1996) and Bhupinderpal-Singh et al. (2003), i.e., that phloem girdling does not deplete NSC in roots completely. This finding supports the view put forward by Millard et al. (2007) and Li et al. (2015) that some NSCs may represent C sequestration rather than storage. Limitation of C remobilization in roots was also found in *Populus tremuloides* seedlings exposed to complete darkness, i.e., under severe C stress (Wiley et al., 2017). That fine root biomass was not significantly different among soil moisture treatments (**Figure 2**) is consistent with the findings of several authors (e.g., Cudlin et al., 2007; Ostonen et al., 2007; Brunner et al., 2009) and the observation of Joslin et al. (2000) that forests maintain a relatively constant fine root biomass over the long-term. These findings indicate that due to rapid turnover fine roots have a high priority for within-tree C allocation and are a substantial sink for plant C (Nadelhoffer and Raich, 1992; Jackson et al., 1997).

In accordance with reports of numerous studies that soil water deficit limits cell division and cell enlargement (Hsiao, 1973; Zweifel et al., 2006; Rossi et al., 2009; Muller et al., 2011; Pantin et al., 2013; Balducci et al., 2015), radial root growth and tracheid diameter in the drought-control treatment were significantly reduced compared to watered trees (**Figure 3**, **Table 1**). The period of root elongation growth was also shortened when low water availability prevailed (Supplementary Figure S1), which is in line with several other studies that root growth ceases under severe drought (for a review see Brunner et al., 2015). However, after girdling root radial growth and CLD of EW was not significantly different among soil moisture treatments indicating that blockage of phloem C transport induced physiological changes that outweighed drought effects imposed on root cambial activity and cell differentiation.

As hypothesized, wood anatomical traits changed in response to girdling and soil water manipulation. The significant increase in CLD (i.e., expansive growth) in the EW of drought-stressed trees was most likely caused by osmotically active sugars formed during starch degradation. However, CLD of EW did not increase in the well-watered treatment (**Table 1**) showing similar soluble sugar content in the bark at the end of the growing season (**Figure 4**). Most likely starch reserves in the xylem tissue, which were not analyzed in this study, were degraded to different extent depending on water availability. Low-molecular-weight sugars are known to decrease osmotic potential and are a key driving variable of cambial activity and cell enlargement in response to girdling (Jones, 1992; De Schepper and Steppe, 2011; Sala et al., 2012; Steppe et al., 2015). According to the Hagen–Poiseuille law, the resulting increase in CLD: CWT ratio in combination with striking increase in EW width enabled girdled trees to transport more water but at higher cavitation risk (Rosner, 2013; Hacke et al., 2015; but see Gleason et al., 2016). An increase in tracheid lumen in the stem was also detected in pine trees in response to drought stress (Martín et al., 2010; Eilmann et al., 2011), which has been suggested to be an adaptation to maximize water uptake when water supply is limited. However, our results are in contrast with the findings of Eldhuset et al. (2013), who reported that tracheid diameter in fine roots of drought-stressed *P. abies* was significantly reduced compared to controls. These contradictory results can be explained by the necessity to mobilize limited C reserves in roots of girdled trees, which triggers the production of less dense wood (increase in CLD: CWT) to reduce formation costs per unit wood volume. The contrasting response in cell enlargement aboveground and belowground in response to girdling, i.e., the decrease of CLD in the tree stem above girdling (Winkler and Oberhuber, 2017) and increase of CLD in coarse roots below girdling (**Table 1**), might be related to accumulation and shortage of some internal hormonal factor(s) above and below the phloem blockage zone, respectively. For example, auxin and cytokinin function as major regulators of cambial activity and cell differentiation (Zhang et al., 2014; Immanen et al., 2016) and are transported acropetally and basipetally through the vascular system (Lacombe and Achard, 2016), which was either blocked (phloem transport) or showed reduced activity (xylem sap flow) in girdled study trees (Oberhuber et al., 2017).

### Phenology of Aboveground and Belowground Growth in Non-girdled Trees

Winkler and Oberhuber (2017) reported that aboveground growth (i.e., radial stem growth and shoot growth) of drought-stressed *P. abies* saplings commenced and ceased in early April and early June, respectively. Here, we report that in those trees root growth in drought-stressed trees started around end of May, i.e., at the time when aboveground growth was largely completed (Supplementary Figure S1). On the other hand, root growth in watered trees started after cessation of shoot growth but continued during vigorous radial stem growth. Because soil temperatures were consistently above 6°C throughout duration of the experiment (**Figure 1**) and lower soil temperatures were found to increasingly limit root growth (Alvarez-Uria and Körner, 2007), our findings support the view that xeric environmental conditions trigger an early shift of C allocation from aboveground growth to the root system (Gruber et al., 2010; Oberhuber et al., 2014). Our interpretation is consistent with an evaluation of a global data set of root to shoot ratios by Ledo et al. (2018), who reported that comparably more biomass is invested belowground with increasing aridity and in saplings and small trees. Furthermore, low and high priority of C allocation to radial stem growth and root growth, respectively (Waring, 1987; Cannell and Dewar, 1994; Ericsson et al., 1996), support our interpretation, but changes in allocation patterns with tree size and age have to be taken into account (Gedroc et al., 1996; Müller et al., 2000). Several authors (Pregitzer et al., 2000; Abramoff and Finzi, 2015; Iversen et al., 2017) have also suggested that internal controls over C allocation are an equally, if not more important driver of root phenology in addition to edaphic or environmental conditions.

## Conclusion

Although prevention of photosynthate transport toward the roots by girdling caused a significant decrease in fine root biomass in both soil moisture treatments, mobilization of starch reserves provoked a striking increase in radial growth and CLD in coarse roots particularly under drought. Hence, we conclude that (i) radial growth and wood formation in coarse roots of *P. abies* saplings are not only dependent on current photosynthates and (ii) phloem girdling induces physiological changes (e.g., concentration of internal growth-regulating factors) that outweigh drought effects imposed on root cambial activity and cell differentiation.

## Author Contributions

WO conceived and designed the experiments and coordinated the research project. GR-L measured and compiled the data. GR-L and WO did the data analysis. WO wrote the manuscript with the contribution from GR-L.

## Conflict of Interest Statement

The authors declare that the research was conducted in the absence of any commercial or financial relationships that could be construed as a potential conflict of interest.
