# Supplementary material for: Phloem Girdling of Norway Spruce Alters Quantity and Quality of Wood Formation in Roots Particularly Under Drought
Source: Front Plant Sci. 2018 Mar 27;9:392. doi: 10.3389/fpls.2018.00392 (PMC5881222; doi:10.3389/fpls.2018.00392)
Supplement: Supplementary file 1 [file Table_1.DOCX]

**Supplementary Table 1** Root radial growth (mean ± standard deviation) in 2014, i.e. in subsets of trees (n = 10) girdled at different dates and exposed to different soil humidity in the experimental year 2015. Statistically significant differences among the control and girdled tree subsets are indicated (* = *P* ≤ 0.05).

|  | Watered subsets | | | | Drought-stressed subsets | | | | |
| --- | --- | --- | --- | --- | --- | --- | --- | --- | --- |
|  | control | GD doy 77 | GD doy 138 | GD doy 190 | | control | GD doy 77 | GD doy 138 | GD doy 190 |
| Earlywood width (µm) | 1104 ± 475 | 1184 ± 915 | 1137 ± 424 | 1353 ± 697 | | 1062 ± 404 | 1353 ± 495 | 1108 ± 723 | 1103 ± 518 |
| Latewood width (µm) | 118 ± 76 | 74 ± 33 | 87 ± 37 | 133 ± 81 | | 163 ± 74 | 85 ± 25***** | 122 ± 62 | 90 ± 55 |
| Ring width (µm) | 1257 ± 533 | 1273 ± 911 | 1225 ± 413 | 1530 ± 692 | | 1240 ± 403 | 1438 ± 493 | 1229 ± 721 | 1193 ± 521 |
