# Supplementary material for: Phloem Girdling of Norway Spruce Alters Quantity and Quality of Wood Formation in Roots Particularly Under Drought
Source: Front Plant Sci. 2018 Mar 27;9:392. doi: 10.3389/fpls.2018.00392 (PMC5881222; doi:10.3389/fpls.2018.00392)
Supplement: Supplementary file 2 [file Image_1.pdf]

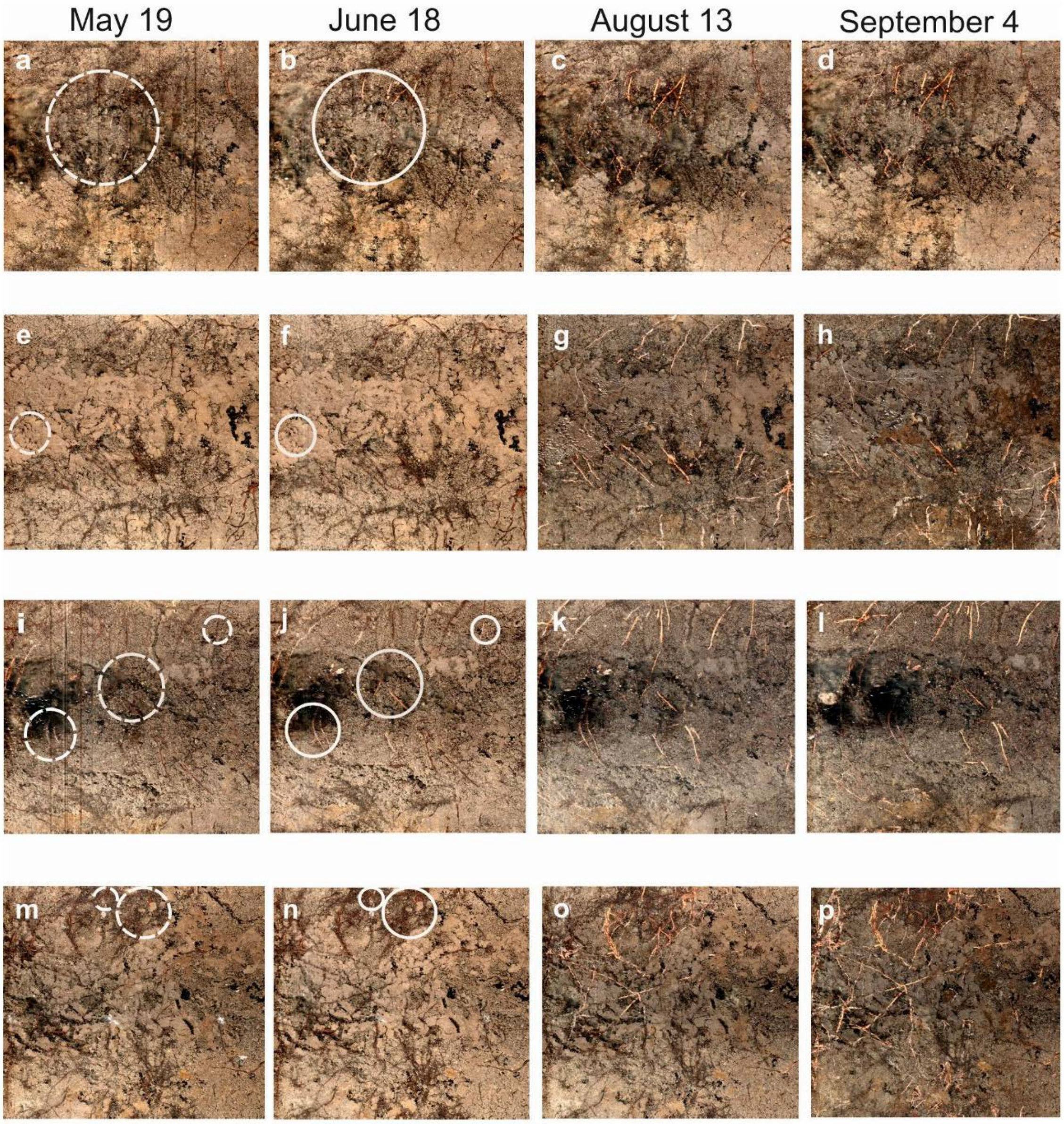

Supplementary Fig. 1a-p Exemplary root scans of drought-stressed control trees (a-d) and drought-stressed trees girdled at GD doy 77 (e-h), GD doy 138 (i-l) and GD doy 190 (m-p) taken on 19 May, 18 June, 13 August and 4 September 2015. Areas circled with continuous lines show the first detected root growth during the growing season 2015 compared to reference area (dashed lines).
